# Molecular prevalence and genetic diversity analysis of *Enterocytozoon bieneusi* in humans in Hainan Province, China: High diversity and unique endemic genetic characteristics

**DOI:** 10.3389/fpubh.2022.1007130

**Published:** 2022-09-06

**Authors:** Tiemin Zhang, Guangxu Ren, Huanhuan Zhou, Yu Qiang, Jiaqi Li, Yun Zhang, Tingting Li, Yunfei Zhou, Yuan Wang, Xiuyi Lai, Shen Lei, Feng Tan, Rui Liu, Wenting Li, Jing He, Wei Zhao, Chuanlong Zhu, Gang Lu

**Affiliations:** ^1^Department of General Surgery, The Second Affiliated Hospital of Hainan Medical University, Haikou, China; ^2^Key Laboratory of Tropical Translational Medicine of Ministry of Education, Hainan Medical University, Haikou, China; ^3^Hainan Medical University-The University of Hong Kong Joint Laboratory of Tropical Infectious Diseases, Hainan Medical University, Haikou, China; ^4^Department of Pathogenic Biology, Hainan Medical University, Haikou, China; ^5^Department of Parasitology, Wenzhou Medical University, Wenzhou, China; ^6^Department of Tropical Diseases, The Second Affiliated Hospital of Hainan Medical University, Haikou, China

**Keywords:** *Enterocytozoon bieneusi*, genotype, human, zoonotic, Hainan

## Abstract

*Enterocytozoon bieneusi* is a zoonotic pathogen commonly found in humans and animals all over the world. Here, we investigated the occurrence and genotype constitute of *E. bieneusi* among the individuals from Haikou city of Hainan, China. A total of 1,264 fecal samples of humans were collected, including 628 samples from patients with diarrhea (325 adults and 303 children) and 636 samples from the asymptomatic population (383 college students and 253 kindergarten children). *E. bieneusi* was detected using nested polymerase chain reaction (PCR) amplification of the internal transcribed spacer (ITS) region. A phylogenetic tree was constructed using a neighbor-joining tree construction method. The overall prevalence of *E. bieneusi* was 3.7% (47/1,264), while it was 5.6% in the patients with diarrhea (5.8% in adults and 5.3% in children) and 1.9% in the asymptomatic population (2.9% in college students and 0.4% in kindergarten children). The prevalence of *E. bieneusi* in humans with diarrhea was significantly higher than that in the asymptomatic population (χ^2^ = 36.9; *P* < 0.05). A total of 28 genotypes were identified, including ten known genotypes: CHG2 (*n* = 3), CHG3 (*n* = 5), CHG5 (*n* = 10), CM21 (*n* = 1), EbpA (*n* = 1), EbpC (*n* = 1), PigEBITS4 (*n* = 1), PigEBITS7 (*n* = 1), SHR1 (*n* = 4), Type IV (*n* = 2), and 18 novel genotypes (HNH-1 to HNH-18; one each). All these genotypes were categorized into three groups, including group 1 (*n* = 6), group 2 (*n* = 14), and group 13 (*n* = 8). This was the first study on the identification of *E. bieneusi* among humans in Hainan, China. The correlation between *E. bieneusi* infection and diarrhea was observed. The high diversity and distinctive distribution of *E. bieneusi* genotypes found in this study reflected the unique epidemic genetic characteristics of *E. bieneusi* in humans living in Hainan.

## Introduction

Phylum Microsporidia is recognized as a group of opportunistic infectious agents worldwide and comprises more than 1,500 species, belonging to 160 genera (1, 2). They are intracellular pathogens, infecting the members of every phylum of the animal kingdom (2). The identification of Microsporidia species in water sources led to their inclusion in the Category B list of biodefense pathogens by the National Institutes of Health (NIH) and microbial contaminant candidates list of concern for waterborne transmission by the Environmental Protection Agency (EPA) (2). To date, a total of 17 species, belonging to the nine genera of Microsporidia, have been identified as opportunistic human pathogens (2). Among them, *Enterocytozoon bieneusi* is the most frequently identified species, which was first found in an AIDS patient in France in 1985 (3). *E. bieneusi*, as an opportunistic pathogen infecting the alimentary tract of hosts, causes a wide spectrum of clinical symptoms in humans, ranging from asymptomatic or self-limiting symptoms in immunocompetent individuals to severe and life-threatening symptoms in the immunocompromised person (4). Humans can acquire *E. bieneusi* infection through several transmission routes, including direct contact with the infected persons (anthroponotic transmission) or animals (zoonotic transmission) and the ingestion of contaminated water or food (4).

*E. bieneusi* is a complex species, having multiple genotypes and diverse host ranges and pathogenicity (4). Many molecular epidemiologic studies have determined the distribution of *E. bieneusi* genotypes in different hosts and have inferred the possible routes of transmission and source of infections (1). To date, more than 685 genotypes of *E. bieneusi* have been identified using PCR analysis of the internal transcribed spacer (ITS) region of the rRNA gene of *E. bieneusi* (1). Among these, at least 33 genotypes have been found in both humans and animals, supporting the presumption of their zoonotic potential (4, 5). On the other hand, all the identified genotypes can be grouped into thirteen different clades, which are named groups 1–13 based on their phylogenetic analysis (6). Groups 1 and 2 are the two largest groups, accounting for 94% of the total genotypes, and are called zoonotic groups (4). These two groups contain almost all the genotypes detected in humans and also contain a vast majority of genotypes from various animal hosts; some genotypes are detected both in humans and animals (4). In contrast, the genotypes, belonging to the remaining 11 groups, are found mostly in the specific hosts and wastewater (4, 7). Understanding the source of *E. bieneusi* infection to cut off its route of transmission is important in adequately controlling its infection in humans due to the lack of effective vaccines and drugs.

Hainan, the only tropical island province in China, has a unique geographical landform, ecological environment, living customs, and culture of Li nationality. It has a wide variety of wild animals and arthropods, thereby having a high incidence of tropical infectious diseases. *E. bieneusi* was primarily detected in the farmed and wild animals in Hainan with a reported prevalence of over 10% (5, 6, 8–10). These data showed that *E. bieneusi* has been widely distributed in Hainan, China. However, the prevalence of this pathogen, causing human infections, has not been reported yet. Therefore, the present study aimed to determine the prevalence and genotypes of *E. bieneusi* among humans in Hainan, China by sequencing and analyzing the ITS region of the rRNA gene. This study also assessed the possible transmission patterns and infection sources of this pathogen by homology and phylogenetic analyses. The results might contribute to developing the control strategies of *E. bieneusi* in Hainan Province, China.

## Materials and methods

### Ethics approval and consent to participate

Approvals for these studies were obtained from the Ethics Committees of Hainan Medical University. Written informed consent was signed by each of adult participants and guardians of minors under 17 years of age after the purposes and procedures of the study were explained to them.

### Specimens

From July 2018 to December 2019, a total of 1,264 fecal samples were collected from Hainan Province of China. Among them, 628 were from patients with diarrhea in the Second Affiliated Hospital of Hainan Medical University, comprising 325 adults and 303 minors. 636 were from asymptomatic people, including 383 college students from Hainan Medical University and 253 children from Shan'gao kindergarten (Table 1). Only one fecal sample per participant was included in the present study. The samples (formed stool: 20 g; watery stool: 30 mL) were placed in a plastic fecal collection box, with collection date, stool characteristics (liquid stool or formed stool) and patient identity (age and gender) being recorded. The collected samples were immediately stored at 4°C and sent to our laboratory for parasite testing within 24 h. The doctors communicated and provided consulting services, a voluntarily participated in the survey and signed a written informed consent form. All the participants did not undergo the antiparasitic treatment.

**Table 1:** Prevalence and distribution of *E. bieneusi* genotypes in humans in Hainan Province of China.

**Clinical symptoms**	**No. positive/No. examined (%)**	**Known genotype (*n*)**	**Novel genotype (*n*)**
**Diarrhea**			
Adults	19/325 (5.8)	CHG5 (4), CHG3 (3), CHG2 (1), EbpA (1), SHR1 (1), Type-IV (1), PigEBITS4 (1), CM21 (1)	HNH-1 to 6 (1 each)
Children	16/303 (5.3)	SHR1 (3), CHG5 (2), CHG2 (2), CHG3 (2), EbpC (1), Type-IV (1)	HNH-7 to 11 (1 each)
Subtotal	35/628 (5.6)	CHG5 (6), CHG3 (5), SHR1 (4), CHG2 (3), Type-IV (2), EbpA, PigEBITS4, CM21, EbpC (1 each)	HNH-1 to 11 (1 each)
**Non-Diarrhea**			
Adults	11/383 (2.9)	CHG5 (4), PigEBITS7 (1)	HNH-12 to 17 (1 each)
Children	1/253 (0.4)	/	HNH-18 (1)
Subtotal	12/636 (1.9)	CHG5 (4), PigEBITS7 (1)	HNH-12 to 18 (1 each)
Total	47/1,264 (3.7)	CHG5 (10), CHG3 (5), SHR1 (4), CHG2 (3), Type-IV (2), EbpC, EbpA, PigEBITS7, PigEBITS4, CM21 (1 each)	HNH 1 to 18 (1 each)

### DNA extraction

Genomic DNA was extracted from ~200 mg of each fecal sample using a QIAamp DNA Stool Mini Kit (QIAgen, Hilden, Germany), following the manufacturer's instructions. In order to obtain a high yield of DNA, the lysis temperature was increased to 95°C according to the manufacturer's suggestions. The extracted DNA samples were eluted in 200 μl of AE buffer and stored at −20°C in a freezer prior to PCR analysis.

### PCR amplification

The presence of *E. bieneusi-*positive DNA was detected using nested PCR amplification of a 410 bp nucleotide fragment of the rRNA gene including 243 bp of the ITS region. The primers and PCR conditions have been previously described (11). TaKaRa Taq DNA Polymerase (TaKaRa Bio Inc., Tokyo, Japan) was used in all the PCR reactions. A negative control without DNA and a positive control with DNA of the *E. bieneusi* BEB6 genotype from goat were included in all the PCR tests. All the secondary PCR products were run on a 1.5% agarose gel and visualized by staining the gel with Goldenview.

### Nucleotide sequencing and analysis

All the secondary PCR products of the expected size were directly sequenced with the same set of primers, which was used for the secondary PCR by Life Technologies (Guangzhou, China), using a Big Dye1 Terminator v3.1 cycle sequencing kit (Applied Biosystems, Carlsbad, CA, USA). The obtained nucleotide sequences were aligned with each other and compared to the reference sequences downloaded from GenBank using the Basic Local Alignment Search Tool (BLAST) (http://www.ncbi.nlm.nih.gov/BLAST/) and ClustalX 1.83 (http://www.clustal.org/) in order to determine the genotypes. According to the established nomenclature system, the nucleotide sequences of the ITS region identical to known genotypes were given the first published name; the nucleotide sequences with single nucleotide substitutions, deletions, or insertions as compared to the known ITS genotypes were considered novel genotypes (12). Meanwhile, the novel genotypes were confirmed by sequencing another two separate PCR products of the same preparations.

### Phylogenetic analysis

In order to better assess the genetic diversity of *E. bieneusi* genotypes in the present study and to determine the genetic correlations of novel genotypes to the known ones, a neighboring-joining phylogenetic tree was constructed using Mega X software (http://www.megasoftware.net/). The phylogenetic tree was based on the evolutionary distances calculated using a Kimura 2-parameter model and analyzed using bootstrap analysis with 1,000 replicates for reliability.

### Statistical analyses

Chi-square analysis was performed to assess the correlations of the prevalence of *E. bieneus*i by group: between diarrheal patients and asymptomatic populations and between adult group and minor group using SPSS (Statistical Package for the Social Sciences) version 17.0. The difference was considered statistically significant when the *P* < 0.05.

### Nucleotide sequence accession numbers

The nucleotide sequences of novel genotypes of *E. bieneusi* obtained in the present study were deposited in the GenBank database under accession numbers: MT193627 to MT193644.

## Results

### Prevalence of *E. bieneusi*

*E. bieneusi* was detected in 3.7% (47/1,264) of the feces samples (Table 1). The *E. bieneusi* prevalence was significantly higher in patients with diarrhea (5.6%) than that in the asymptomatic populations (1.9%) (χ^2^ = 36.9; *P* < 0.05). Different prevalences of *E. bieneusi* were observed between adults and minors: 5.8 and 5.3% in diarrheal patients and 2.9 and 0.4% in asymptomatic populations. However, by χ^2^-test, significant difference in prevalence was only observed in college students and kindergarten children (χ^2^ = 5.6; *P* = 0.02).

### Characterization and distribution of *E. bieneusi* genotypes

The sequence analysis of 47 *E. bieneusi* isolates obtained in this study identified a total of 28 genotypes, including ten known genotypes (CHG2, CHG3, CHG5, CM21, EbpA, EbpC, PigEBITS4, PigEBITS7, SHR1, and Type IV) and 18 novel genotypes (HNH-1 to HNH-18). Among them, CHG5 accounted for the largest proportion (21.3%, 10/47), followed by CHG3 (10.6%, 5/47), SHR1 (8.5%, 4/47), CHG2 (6.4%, 3/47), Type IV (4.3%, 2/47), and the remaining 23 genotypes (each 2.1%, 1/47). Genotype distribution of *E. bieneusi* in different groups could be seen in Table 1. Genotype CHG5 was the most widely distributed in humans in the investigated areas, which was detected in both the diarrhea patients and the asymptomatic populations. Meanwhile, result of the homology analysis of the novel genotypes of *E. bieneusi* showing in Table 2.

**Table 2:** Homology analysis of the novel genotypes of *E. bieneusi* identified in this study.

**Genetic group**	**Genotype (accession no)^a^**	**Genotype (accession no)^b^**	**Nucleotide (site)**
Group 1	HNH-14 (MT193640)	EbpC (MH024028)	Insertions T (52); G (53)
Group 2	HNH-1 (MT193627)	CHG3 (KP262362)	C → T (232)
	HNH-4 (MT193630)		A → G (164)
	HNH-10 (MT193636)		A → G (108) and T → A (182)
	HNH-13 (MT193639)		T → C (99)
	HNH-17 (MT193643)		T → C (79)
	HNH-18 (MT193644)		C → T (153)
	HNH-6 (MT193632)	CHG2 (KP262366)	G → A (82)
	HNH-9 (MT193635)		T → A (172)
	HNH-11 (MT193637)	CHG5 (KP262365)	G → T (210)
	HNH-16 (MT193642)		C → T (232)
Group 13	HNH-2 (MT193628)	SHR1 (MN523336)	T → A (52)
	HNH-3 (MT193629)		T → C (111)
	HNH-5 (MT193631)		A → G(101)
	HNH-7 (MT193633)		T → C (213)
	HNH-8 (MT193634)		T → C (99)
	HNH-12 (MT193638)		A → G (71); T → C (105)
	HNH-15 (MT193641)		Deletion A (143)

^a^Accession nos. of the novel ITS genotypes obtained in this study.

^b^Accession nos. of the known genotypes having the largest homology with the novel ones obtained in this study.

### Phylogenetic analysis

**Figure 1:**
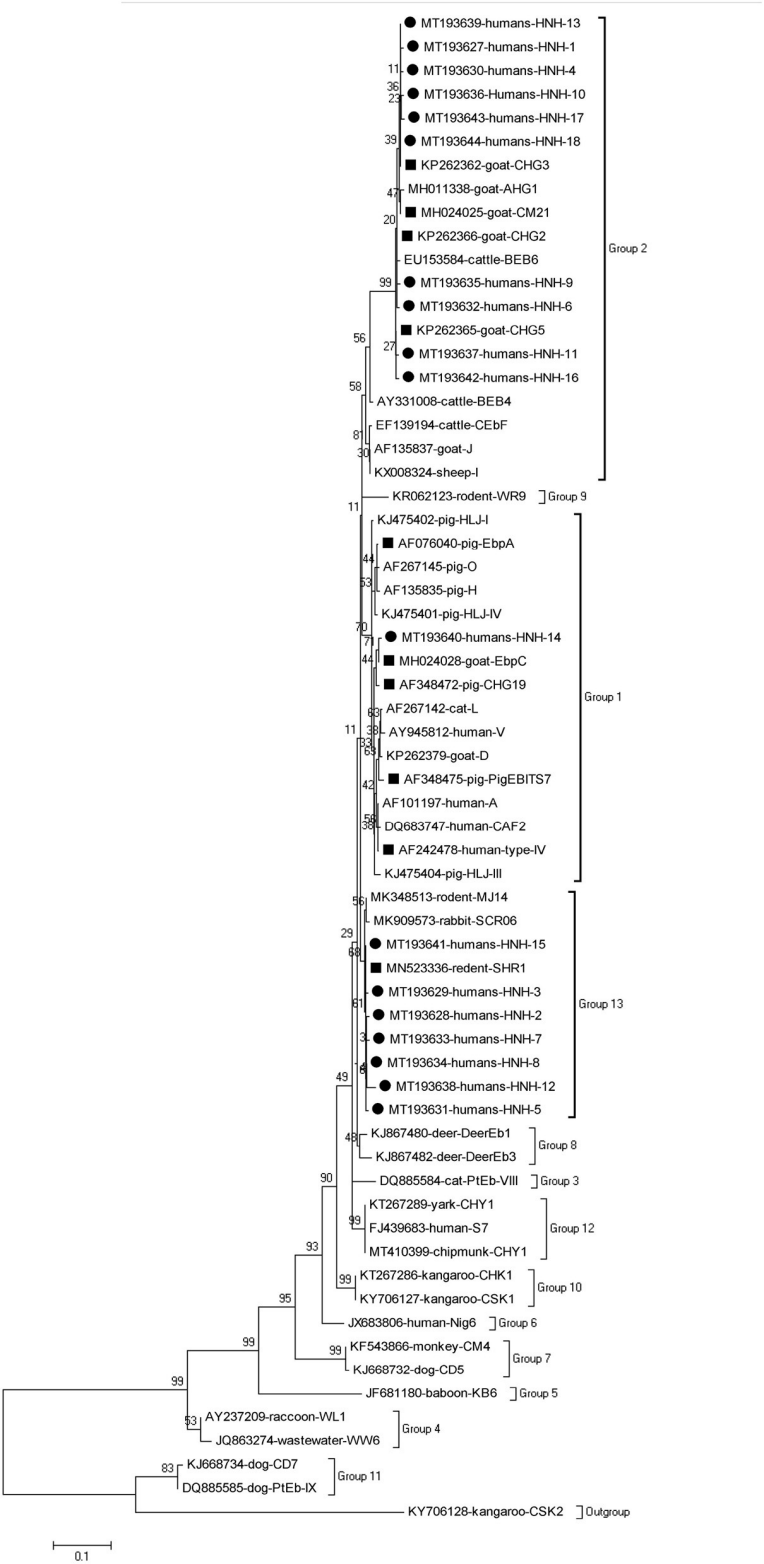
Phylogenetic relationship of the genotypes of *E. bieneusi* in humans. The tree was constructed by a neighboring-joining phylogenetic analysis of ITS sequences using the Kimura-2-parameter model and with 1,000 replicates. Each sequence is identified by its accession number, host origin, and genotype designation. The square and circle filled in black indicate known and novel genotypes, respectively.

The phylogenetic analysis of the ITS region of *E. bieneusi* divided the genotypes, which were identified in humans in this study, into three groups. Five known genotypes (EbpA, EbpC, PigEBITS4, PigEBITS7 and Type IV) and one novel genotype (HNH-14) were categorized into group 1. Four known genotypes (CM21, CHG5, CHG3, and CHG2) and ten novel genotypes (HNH-1, HNH-4, HNH-6, HNH-9 to HNH-11, HNH-13, and HNH-16 to HNH-18) were categorized into group 2. The remaining one known genotype (SHR1) and seven novel genotypes (HNH-2, HNH-3, HNH-5, HNH-7, HNH-8, HNH-12, and HNH-15) were categorized into group 13 (Figure 1).

## Discussion

Since its first report on humans in China in 2011, a total of 14 epidemiological studies on the prevalence of *E. bieneusi* have been performed in ten provinces/municipalities of China, including Xinjiang, Yunnan, Shanghai, Henan, Heilongjiang, Jilin, Chongqing, Shandong, Guangxi, and Hubei Provinces (13–16). The highest prevalence was 22.5% in Changchun (17) and the lowest prevalence was 0.2% in Wuhan (18). The overall prevalence was 6.4, 8.1, and 3.6% among diarrhea patients, HIV patients, and healthy individuals in China, respectively (15). In the present study, a total of 3.7% (47/1,264) of the human fecal samples from Hainan province were tested positive for *E. bieneusi* using PCR and sequencing analysis of the ITS gene region. Overall, this prevalence was lower than the average prevalence, especially among the kindergarten children with diarrhea, among whom, the prevalence was only 5.3%, which was not higher than that in the adults. Previous studies have shown that the prevalence of *E. bieneusi* was high up to 78% in children in Uganda (19). About 22.5% of diarrhea infection in children was caused by *E. bieneusi* in northeast China (17), which might be due to the regional differences because, in Hainan, the kindergarten children are less exposed to animals due to the fewer pets in the families.

In the present study, the prevalence of *E. bieneusi* in diarrhea patients (5.6%) was significantly higher than that in the asymptomatic population (1.9%) (χ^2^ = 36.9; *P* < 0.05). In China, two previous studies revealed significantly higher prevalence of *E. bieneusi* in children with diarrhea than in children without diarrhea: 4.9% (11/223) vs. 3.7% (13/350) in Shanghai (20); 25.0% (1/4) vs. 7.2% (18/251) in Heilongjiang (21). Besides, researchers from other study also reported that children with diseases or diarrhea were more susceptible to *E. bieneusi* infection (22). Therefore, *E. bieneusi* infection might be an important factor causing diarrhea. Therefore, it is necessary to monitor the infection of *E. bieneusi* among patients with diarrhea especially in the immunocompromised populations in order to avoid patient death due to misdiagnosis.

Among the known genotypes identified in this study, the genotypes Type-IV, EbpC, EbpA, and PigEBITS7 have been previously reported in humans and animals in several provinces of China and other countries (4). The remaining six known genotypes (CHG2, CHG3, CHG5, CM21, PigEBITS4, and SHR1) and 18 novel genotypes (HNH-1 to HNH-18) were reported for the first time in humans. This not only enriched the *E. bieneusi* genotypes, which can infect humans, and expanded the host range of these genotypes but also suggested that the ruminant-specific genotypes (CHG2, CHG3, and CHG5) could also infect humans. Therefore, the zoonotic risk of these genotypes should also be reevaluated. Actually, the 10 known genotypes (CHG2, CHG3, CHG5, CM21, EbpA, EbpC, PigEBITS4, PigEBITS7, SHR1, and Type IV) were all identified in a large number of farm animals in Hainan province (5, 6, 8–10). Therefore, it was speculated that these genotypes, infecting humans in this region, might have come from animals.

In this study, the 18 novel genotypes identified belonged to three groups: genotype HNH-14 in group 1; genotypes HNH-1, HNH-4, HNH-6, HNH-9 toHNH-11, HNH-13, and HNH-16 to HNH-18 in group 2; genotypes HNH-2, HNH-3, HNH-5, HNH-7, HNH-8, HNH-12, and HNH-15 in group 13. Prior to this study, a total of 66 genotypes have been identified in humans, among which, 48, 12, 2, and 4 genotypes belong to Groups 1, 2, 5, and 6, respectively (1, 4). The genotypes identified in humans, belonging to Group 1, might have zoonotic potential due to their close relation to a wide range of hosts and the lack of geographic segregation among humans (4). The identification of the previously considered ruminant-adapted group 2 genotypes (notably BEB4, BEB6, CHN3, I, and J) in the humans, residing in the Czech Republic and China, indicated a possibility of zoonotic transmission for these genotypes (4, 17, 23). The genotypes in groups 5, 6 had a relatively narrow host range and robust geographic specificity because the genotypes, such as Nig3, Nig4, Nig6, and Nig7, have been identified in humans only in Africa (24, 25). The fact of 50% (14/28) and 28.6% (8/28) of the genotypes of groups 2 and 13, respectively, revealed unique epidemic genetic characteristics of *E. bieneusi* in humans living in Hainan, China.

In conclusion, this was the first study on the identification of *E. bieneusi* in humans in Hainan, China. The prevalence of *E. bieneusi* was 3.7% (47/1,264). The prevalence of *E. bieneusi* in humans with diarrhea was significantly higher than that in the asymptomatic population. The high diversity and distinctive distribution of *E. bieneusi* genotypes found in this study reflected the unique epidemic genetic characteristics of *E. bieneusi* in humans living in Hainan.

## Data availability statement

The datasets presented in this study can be found in online repositories. The names of the repository/repositories and accession number(s) can be found in the article/supplementary material.

## Ethics statement

Approval for these studies were obtained from the Ethics Committee of Hainan Medical University. Written informed consent was signed by each participant (for the 17-year-old participants, the consent forms were obtained from their parents or guardian) after they or their parents or guardian were informed of the purposes and procedures of the study.

## Author contributions

WZ, GL, and CZ contributed to the study conceive and design. TZ, RL, WL, JH, and CZ contributed to acquisition of clinical sample. GR, HZ, YQ, JL, YZha, TL, YZho, YW, XL, and SL performed experiments. TZ, GR, HZ, and WZ contributed to acquisition of clinical data. GR and WZ contributed to statistical analysis. WZ contributed to writing the manuscript. GL obtained funding. FT and CZ provided administrative, technical support, and constructive discussion. All authors approved of the final version to be published and agree to be accountable for all aspects of the manuscript.

## Funding

This work was supported by the National Natural Science Foundation of China (82060375 and 81760378), Research Project of Hainan Academician Innovation Platform (YSPTZX202004), Major Science and Technology Program of Hainan Province (ZDKJ202003), Hainan Talent Development Project (SRC200003), Open Foundation of Key Laboratory of Tropical Translational Medicine of Ministry of Education, Hainan Medical University (2020TTM004), and Hainan Province Clinical Medical Center.

## Conflict of interest

The authors declare that the research was conducted in the absence of any commercial or financial relationships that could be construed as a potential conflict of interest.

## Publisher's note

All claims expressed in this article are solely those of the authors and do not necessarily represent those of their affiliated organizations, or those of the publisher, the editors and the reviewers. Any product that may be evaluated in this article, or claim that may be made by its manufacturer, is not guaranteed or endorsed by the publisher.
